# Pentosan polysulfate binds to STRO-1^+^ mesenchymal progenitor cells, is internalized, and modifies gene expression: a novel approach of pre-programing stem cells for therapeutic application requiring their chondrogenesis

**DOI:** 10.1186/s13287-017-0723-y

**Published:** 2017-12-13

**Authors:** Jiehua Wu, Susan Shimmon, Sharon Paton, Christopher Daly, Tony Goldschlager, Stan Gronthos, Andrew C. W. Zannettino, Peter Ghosh

**Affiliations:** 1 Proteobioactives Pty. Ltd., PO Box 174, Balgowlah, Sydney, New South Wales 2093 Australia; 2 Myeloma Research Laboratory, Faculty of Health and Medical Sciences, University of Adelaide and the Cancer Theme, South Australia Health and Medical Research Institute, Adelaide, South Australia 5000 Australia; 3 Department of Surgery, Monash University, Clayton, Victoria 3168 Australia; 4 Department of Neurosurgery, Monash Medical Centre, Clayton, Victoria 3168 Australia; 5 The Ritchie Centre, Hudson Institute of Medical Research, Monash University, Clayton, Victoria 3168 Australia; 6 Mesenchymal Stem Cell Laboratory, Adelaide Medical School, Faculty of Health and Medical Sciences, University of Adelaide, Adelaide, South Australia 5000 Australia; 7 Present address: Minomic International Ltd, Suite 2, 75 Talavera Rd, Macquarie Park, NSW 2113 Australia; 8 Present address: School of Mathematical and Physical Sciences, Faculty of Science, University of Technology Sydney, Broadway, PO Box 123, Sydney, NSW 2007 Australia

**Keywords:** Mesenchymal progenitor cells, Pentosan polysulfate, Heparin, Chondrogenesis, Proliferation, Gene expression, CD146

## Abstract

**Background:**

The pharmaceutical agent pentosan polysulfate (PPS) is known to induce proliferation and chondrogenesis of mesenchymal progenitor cells (MPCs) in vitro and in vivo. However, the mechanism(s) of action of PPS in mediating these effects remains unresolved.

In the present report we address this issue by investigating the binding and uptake of PPS by MPCs and monitoring gene expression and proteoglycan biosynthesis before and after the cells had been exposed to limited concentrations of PPS and then re-established in culture in the absence of the drug (MPC priming).

**Methods:**

Immuno-selected STRO-1^+^ mesenchymal progenitor stem cells (MPCs) were prepared from human bone marrow aspirates and established in culture. The kinetics of uptake, shedding, and internalization of PPS by MPCs was determined by monitoring the concentration-dependent loss of PPS media concentrations using an enzyme-linked immunosorbent assay (ELISA) and the uptake of fluorescein isothiocyanate (FITC)-labelled PPS by MPCs. The proliferation of MPCs, following pre-incubation and removal of PPS (priming), was assessed using the Wst-8 assay method, and proteoglycan synthesis was determined by the incorporation of ^35^SO_4_ into their sulphated glycosaminoglycans. The changes in expression of MPC-related cell surface antigens of non-primed and PPS-primed MPCs from three donors was determined using flow cytometry. RNA sequencing of RNA isolated from non-primed and PPS-primed MPCs from the same donors was undertaken to identify the genes altered by the PPS priming protocol.

**Results:**

The kinetic studies indicated that, in culture, PPS rapidly binds to MPC surface receptors, followed by internalisation and localization within the nucleus of the cells. Following PPS-priming of MPCs and a further 48 h of culture, both cell proliferation and proteoglycan synthesis were enhanced. Reduced expression of MPC-related cell surface antigen expression was promoted by the PPS priming, and RNA sequencing analysis revealed changes in the expression of 42 genes.

**Conclusion:**

This study has shown that priming of MPCs with low concentrations of PPS enhanced chondrogenesis and MPC proliferation by modifying their characteristic basal gene and protein expression. These findings offer a novel approach to re-programming mesenchymal stem cells for clinical indications which require the repair or regeneration of cartilaginous tissues such as in osteoarthritis and degenerative disc disease.

**Electronic supplementary material:**

The online version of this article (doi:10.1186/s13287-017-0723-y) contains supplementary material, which is available to authorized users.

## Background

Adult mesenchymal stem cells (MSCs) are an abundant source of self-renewing, multipotent undifferentiated cells that can be readily isolated from bone marrow, adipose tissue, muscle, and synovium. They can be serially expanded in culture and cryopreserved almost indefinitely without significant loss of their tissue regenerative capacity [1–4]. In-vitro studies have shown that when MSCs are exposed to the appropriate physical, chemical, or biological stimuli they will differentiate into cells of the mesodermal lineage, including osteoblasts, chondrocytes, tenocytes, myocytes, and adipocytes [3–5]. Moreover, when administered systemically, MSCs exhibit the capacity to migrate to the site(s) of tissue injury, where they can modulate inflammatory and immune-regulatory pathways as well as release pro-anabolic factors [6–9]. These unique activities of MSCs have led to extensive investigations into their potential applications as biological agents for the treatment of a variety of clinical applications [5–7]. MSCs have been considered a suitable therapy for muscular skeletal and connective tissue disorders, including degenerative disc disease, osteoarthritis, and repair of articular cartilage, owing to the high incidence of such disorders as well as their limited capacity for spontaneous repair and the limited treatment options [10–16].

As indicated, MSCs possess the ability to localize to sites of tissue injury, suppress inflammation, and facilitate repair. Moreover, there is considerable evidence to suggest that MSCs engraft at these sites, undergo differentiation, and synthesise an extracellular matrix consistent with the endogenous tissue [17, 18]. However, for the regeneration or repair of cartilaginous tissues it is important that the initial differentiation of MSCs to chondrocytes is not followed by further differentiation to osteoblasts, a process that has been observed in some experimental studies using these osteochondral precursors [19, 20].

In previous studies [21] we showed that the incubation of STRO-1^+^ immuno-selected mesenchymal progenitor cells (MPCs) with the pharmaceutical agent pentosan polysulfate (PPS) not only improved their viability and enhanced their chondrogenic differentiation but also suppressed osteogenesis in vitro. In subsequent in-vivo studies using ovine models, MPCs were formulated with PPS and injected directly into degenerate intervertebral discs, and were found to promote the deposition of a new disc matrix without evidence of osteogenic differentiation [22–24]. However, in these animal studies the MPCs and PPS were always mixed together immediately prior to administration. As such, it remained to be determined whether the positive outcomes observed represented the sum of the pharmacological activities of the individual components or whether the mechanism of action was via a reprogramming of MPC genetic expression mediated by PPS.

The objective of the present study was to address this question by examining the concentration-dependent binding and internalization of PPS by MPCs and determine if priming of the cells with the drug changed their genetic signature.

## Methods

### Preparation of human STRO-1^+^ immuno-selected mesenchymal progenitor stem cells

Bone marrow was collected from the posterior iliac crest of healthy volunteers (20–35 years old) following their informed consent; the procedure was approved by the Human Research Ethics Committee of the Royal Adelaide Hospital (RAH), Adelaide, South Australia. These aspirates were used to prepare immuno-selected STRO-1^+^ MPCs employing procedures described previously [25]. Briefly, STRO-1^+^ mesenchymal precursor cells derived from the bone marrow aspirates were isolated by STRO-1 magnetic activated cell sorting and used to establish primary cultures. The primary cultures were expanded by trypsin-EDTA detachment and re-plating at a density of 4.0 × 10^4^ cells per cm^2^ as previously described [25]. Following 3–4 passages, the cells were harvested by trypsin-EDTA detachment and re-suspended in culture medium at a density of 5.0–20 × 10^6^ cells/ml. They were then combined with ProFreeze-CDM NAO freezing medium (Lonza Australia Ltd., Blackburn Rd., Mt Waverley, Victoria 3149, Australia) (2×) containing DMSO (7.5%), they were control-rate cryopreserved and placed at –80 °C overnight, and subsequently transferred to the vapour phase of liquid nitrogen until required.

### Competitive PPS enzyme-linked immunosorbent assays (ELISAs) of culture media

The concentration of PPS in culture media was determined with a competitive ELISA using a biotinylated monoclonal antibody (1B1) against polysulphated polysaccharides (kindly provided by Professor Prachya Kongtawelert, Department of Biomedical Sciences, Chiang Mei University, Thailand).

Each well of a 96-well plate was coated with 100 μl 50 μg/ml hexadimethrine bromide (Polybrene; Sigma-Aldrich, Sydney, Australia)) in phosphate-buffered saline (PBS), pH 7.4, and incubated at 37 °C for 1 h. The solution was aspirated and the plate was air-dried without washing. Wells were then blocked with 200 μl/well blocking solution (PBS + 1% bovine serum albumin (BSA)) and incubated at 37 °C for 1 h. The solution was aspirated and the wells were washed with 300 μl/well PBST (PBS + 0.05% Tween-20) three times. The plates were flicked to remove the contents of the wells and dried. The monoclonal antibody B1B1 was diluted 1:200 in Dulbecco’s modified Eagle’s medium (DMEM) and used as the primary antibody solution. The PPS compound (BenePharmachem, Munich, Germany) was used to prepare a 1 mg/ml working stock and was subsequently diluted in DMEM to create a standard curve of 0.004–4 μg/ml. The PPS standard solutions were each mixed with the 1B1 antibody solution in a 1:1 ratio and incubated at 37 °C for 1 h. Aliquots of the inhibition mixtures (100 μl) were transferred to each well and incubated at 37 °C for a further 1 h. Using the same plates, culture media samples containing PPS were mixed 1:1 with the 1B1 antibody solution in the microtitre plate wells. The samples were aspirated from each well and the plate washed with 300 μl/well PBST three times, flicked, and dried. Monoclonal anti-biotin-alkaline phosphatase (AP) antibody (Sigma-Aldrich, Sydney, Australia, cat. no. A-6561) was used as the secondary antibody and was diluted 1:5000 with blocking solution and 100 μl added to each well followed by incubation at 37 °C for 1 h. The antibody solution was aspirated and the plate was washed with 300 μl/well PBST three times, flicked, and dried. The AP substrate, para-nitrophenyl phosphate (PNP; 200 μl 1 mg/ml PNP in 0.1 M NaHCO_3_ buffer containing 2 mM MgCl_2_, pH 8.6) was added to each well and the plates incubated in the dark for 20 min. Absorbance at 405 nm was then determined with a micro-plate reader. All assays were performed in triplicate.

### Kinetics of PPS uptake by MPCs in culture

Primary MPC monolayers were established in culture as described previously [21]. Briefly, 3.0 × 10^5^ MPCs were seeded into wells of 48-well plates and incubated with DMEM containing 10% fetal bovine serum (FBS) at 37 °C in 5% CO_2_ for 16 h. The media from the primary cultures was discarded and the wells were washed with DMEM (3 × 500 μl/well); media and washings were discarded and then replaced with DMEM (500 μl/well) containing gradient concentrations of PPS (0.5, 1.0, 2.5, 5, and 10 μg/ml/well). Plates were maintained at 37 °C in 95% air/CO_2_ and, after 0.25, 0.50, 2, 6, 20, and 24 h, media from individual wells were aspirated, cells washed (PBS, 0.5 ml/well) and media and washings pooled. The concentrations of PPS remaining in the aspirated media and washings of the cultures at each time point was determined using the PPS ELISA as described above.

### The preparation of fluorescein isothiocyanate (FITC)-labelled PPS

PPS (100 mg) was converted to the tetrabutyl ammonium (TBA) salt by incubating with tetrabutylammonium bromide (100 mg; Sigma-Aldrich, Sydney, Australia) dissolved in 10 ml de-ionized H_2_O for 4 h at ambient temperature. The PPS-TBA complex was dialyzed against de-ionized water for 24 h to remove excess salts and then lyophilized. The PPS-TBA complex (50 mg, dissolved in 1 ml DMSO) was mixed with 1,1-carbonyl di-imidazole (28.0 mg/0.5 ml; Sigma-Aldrich, Sydney, Australia)) and incubated at 56 °C for 1 h. After cooling to room temperature, hydrazine (47.8 mg; Sigma-Aldrich, Sydney, Australia) was added and the solution incubated with shaking for 16 h at 45 °C. The PPS carboxyhydrazide complex was then reacted with FITC (Sigma-Aldrich, Sydney, Australia) using the manufacturer’s instructions to convert the FITC-PPS derivative into a TBA salt derivative. The PPS-FITC-TBA salt was then converted to the sodium salt by mixing at 4 °C with 4.0 M NaCl (100 ml) for 16 h followed by 48 h dialysis against water with changes every 16 h, and then lyophilized. The lyophilized PPS-FITC derivative was purified by size-exclusion chromatography on a Superdex-200 column (GE Healthcare Ltd., Sydney, Australia) equilibrated in 0.25 molar NaCl. Column fractions were monitored for PPS concentration using the dimethyl-methylene blue assay [26] and FITC by fluorescence excitation/emission at 485/538 nm. Fractions positive for PPS and FITC fluorescence were pooled, desalted, and lyophilized. The purity of the PPS-FITC complex was established by NMR spectroscopy (by Dr. Ronald Shimmon, Department of Chemistry, University of Technology, Sydney, Australia).

### PPS-FITC uptake by MPCs using fluorescence microscopy

#### Fluorometric assay

Primary MPC monolayer cultures were established in six-well plates (2.5 × 10^5^ cells/well) as described previously [21]. After 16 h, DMEM (3 ml) containing various concentrations of PPS-FITC (0, 1.0, 2.5, 5.0, 10.0, and 20.0 μg/ml) were added to the wells and incubated at 37 °C in 5% CO_2_ for a further 24 h. The media were collected from each well, and the cells were washed 3× with PBS at room temperature. Media and washings were discarded. The washed cells were released from the plates with 250 μl 0.25% trypsin/EDTA at 37 °C for 10 min. The cells and supernatant were separated by centrifugation at 500 g for 10 min, the supernatants were discarded, and the cell pellets washed 3× with PBS (1 ml/well). The cell pellets derived from each culture well were re-suspended in 100 μl de-ionised H_2_O then transferred to wells of black microplates. The microplates were agitated for 1 h to lyse the cells in the absence of light, and the intensity of the fluorescence emission at 538 nm determined for all added PPS-FITC concentrations using a fluorescence microplate reader (Labsystems Fluoroskan II, ThermoFisher Scientific Australia Pty. Ltd., Scoreby, Australia) with de-ionised H_2_O as a blank. The levels of PPS-FITC in each well were quantified using a standard curve prepared from the purified PPS-FITC prepared above.

#### Qualitative assay

MPCs (6000 cells/well) were seeded on eight-well slides (Lab-Tek-II® Chamber Slide System, Permanox®, Grand Island, NY, USA) and incubated at 37 °C in a 5% CO_2_ atmosphere for 16 h. DMEM media (1.0 ml) containing 0, 0.5, 1.0, and 2.5 μg/ml PPS-FITC was added to each well and the slides incubated for a further 24 h at 37 °C. The media were removed and the bound cells washed 3× with 1.0 ml PBS. Media and washings were discarded and cells fixed using 300 μg/well HistoChoice MB Fixatives (Amresco, Solon, OH, USA) for 20 min at room temperature. After washing once with PBS, the cells of each slide were stained with 20 μg/ml propidium iodide (PI) for 10 min at room temperature, washed 3× with PBS, once with 70% ethanol, and 3× with absolute ethanol, and then viewed under UV light using a Nikon Eclipse 80 fluorescence microscopic (Coherent Scientific, Hilton, Australia). Cells were viewed for FITC and PI fluorescence using excitation and emission wavelengths of 485/538 nm for 2 s and 535/620 nm for 60 s, respectively. The cell images were captured using a digital camera coupled to the microscope and images analysed using the NIS-Elements software (Coherent Scientific, Hilton, Australia).

### Assessment of MPC proliferation alone and after priming with PPS

Triplicate cultures of passage 4 MPCs at densities of 1 × 10^6^ cells/ml were established in 24-well plates as described previously [21]. High-glucose DMEM containing 5 μg/ml PPS was then added to 12 wells of the plates and an equivalent volume DMEM alone to the remaining wells. After incubation for 24 h, media were removed from all wells and cells were washed 3× with PBS and then re-established in culture. After 4, 24, and 48 h, incubations were stopped, media removed and cells washed 3× with PBS; media and washings were then discarded. Cells were released from the plates by trypsin/EDTA treatment, the harvested cells from each well were re-suspended in PBS, and aliquots were then analysed to determine MPC proliferation for each of the culture time periods using a commercial cell counting kit (Wst-8 Kit (CCK-8); Sigma-Aldrich, Sydney, Australia) according to the manufacturer’s instructions. As the non-PPS primed MPC cultures failed to demonstrate significant variation in their proliferation over the three time periods, the values obtained from each incubation period were pooled and used as the non-PPS pre-treatment control.

### Proteoglycan synthesis by MPCs alone and after priming with PPS

Wells of six-well culture plates were seeded with passage 4 MPCs (2.8 × 10^5^/well) and incubated with DMEM + 10% FBS at 37 °C in 5% CO_2_ for 16 h. High-glucose DMEM containing 5 μg/ml PPS was then added to three wells of the plates and DMEM alone to the remaining three wells. After incubation for 24 h, media were removed from all wells and cells were washed twice with PBS (3 ml/well) and then re-established in culture. The biosynthesis of proteoglycans (PGs) by these cells over 24 h was then determined as previously described [21]. Briefly, media (3 ml) containing 2.2 μCi/ml H_2_^35^SO_4_ (Perkin-Elmer Life and Analytical Science Knoxfield, Victoria, Australia) was added to each well and plates incubated for 48 h. The medium was removed and discarded. Cells were washed with 3× PBS, and then collagenase solution (Sigma Aldrich, Sydney, Australia; 500 μl, 1 mg/ml) was added to each well and the plate incubated at 37 °C for 1 h to detach the cells and matrix from the plates. The collagenase digests were transferred to 1.5-ml tubes and an equal volume of acetate-buffered papain (Sigma-Aldrich, Sydney, Australia; 1 mg/ml) added to each tube. After incubation at 65 °C for 1.5 h, aliquots (100 μl) of the digests were assayed for DNA content [27] and the remainder transferred to 1.5-ml tubes, and 40 μl 1 mg/ml chondroitin sulphate A (Sigma Aldrich, Sydney, Australia) and 60 μl 5% aqueous cetyl pyridinium chloride (CPC; Sigma Aldrich, Sydney, Australia) was added. The tubes were vortexed and then centrifuged at 11,000 rpm for 3 min to pellet the precipitated ^35^S-glycosaminoglycan (GAG)-CPC complex. The precipitates were collected by centrifugation, washed (3× PBS), and then dissolved in 1 ml scintillant (Perkin-Elmer Life and Analytical Science Knoxfield, Victoria, Australia) and transferred to a scintillation vial. The radioactivity of ^35^S incorporated to newly synthetized S-GAGs of the PGs was determined by scintillation counting (Perkin-Elmer Tricarb 2910TR, Perkin-Elmer Corp., Massachusetts, USA). Results were calculated as ^35^S-GAG-DPM/μg DNA as an index of proteoglycan synthesis per cell.

### Monitoring of MPC phenotypic receptors by flow cytometry

Suspensions of passage 4 MPCs (2.5 × 10^5^) derived from three independent healthy young donors (RAH1, RAH2, and RAH3) were seeded into each well of a six-well plate (in duplicate) and incubated with DMEM + 10% FBS at 37 °C in 5% CO2 for 16 h. The next day, DMEM containing 5 μg/ml PPS was added to three wells of both six-well plates. The remaining three wells of the same plates only received DMEM and were used as the controls (MPCs alone). After an additional 24 h, the cultures from one plate were terminated. The remaining plate was incubated for a further 24 h (i.e. a total incubation time of 48 h). At termination, all media were removed and the six wells of the plates were washed twice with PBS (3 ml/well). Media and washings were discarded, and MPCs were detached from wells by trypsin/EDTA treatment; enzyme activity was quenched and the cells were strained through a 70-μm cell strainer (Becton Dickinson Biosciences, CA, USA) to ensure preparation of single cell suspensions. The MPC suspensions were washed with 10 ml wash buffer (Hank’s buffered salt solution + 5% fetal calf serum (FCS)) and then centrifuged at 400 *g* for 7 min at 4 °C. Cells were re-suspended in blocking buffer (wash buffer supplemented with 1% (v/v) normal human serum + 1% v/v BSA) and counted in 0.4% Trypan Blue and left on ice in blocking buffer for 30 min. Cells were then pelleted by centrifugation (400 g for 7 min at 4 °C), and the supernatant removed and discarded. The cell pellet was re-suspended in 100 μl of one of the primary antibody listed in Table 1 at a final concentration of 20 μg/ml per tube or 100 μl neat supernatant antibody. After maintaining the tubes at 4 °C for 45–60 min, cells were washed twice with 2 ml cold wash buffer and centrifuged at 400 g for 7 min at 4 °C. Cells were re-suspended in 100 μl blocking buffer containing the appropriate secondary goat anti-mouse antibody or FITC-conjugated antibody at a 1:50 dilution (Southern Biotechnology, USA) (Table 1) and incubated for 30 min and then washed twice with 2 ml cold wash buffer at 400 g for 5 min at 4 °C. Antibody-labelled MPCs were then re-suspended in 0.5 ml FACS FIX (1% (v/v) formalin, 0.1 M d-glucose, 0.02% sodium azide, in PBS) for flow cytometric analysis using a BD FACS Canto II and Flow Data Analysis Software V10 (Becton Dickinson Biosciences, CA, USA).

**Table 1:** Primary and secondary antibodies used for MPC ± PPS cytometric analysis

Primary antibodies	Type	Origin
Stro-1	In-house antibody	Provided by Prof. S. Gronthos and Prof. A. Zannettino
CD73	Purified mouse anti-human CD73	BD Pharmingen 550256
CD90	Biotin mouse anti-human CD90	BD Pharmingen 555594
CD105	Purified mouse anti-human CD105	BD Pharmingen 555690
CD44 (H9H11)	In-house antibody	Provided by Prof. S. Gronthos and Prof A. Zannettino
CD146 (CC9)	In-house antibody	
CD34	CD34 FITC	Beckman Coulter IM1870
CD45	CD45 FITC	Beckman Coulter IM0782U
CD14	CD14 FITC	Beckman Coulter IM0645U
Secondary antibodies		
Streptavidin FITC conjugate		Invitrogen SA1001
IgM FITC	Goat anti-mouse IgM FITC	Southern Biotech 1020-02
IgG FITC	Goat anti-mouse IgG FITC	Southern Biotech 1030-02
Negative Controls		
IgM	1A6.12 isotype-matched negative control/anti-salmonella	Provided by Dr. L Ashman
IgG1	1B5 isotype-matched negative control/anti-salmonella	Provided by Dr. L Ashman
IgG2a	1D4.5 isotype-matched negative control/anti-salmonella	Provided by Dr. L Ashman

*CD* cluster differentiation, *FITC* fluorescein isothiocyanate, *Ig* immunoglobulin

### Extraction of RNA from MPC cultures and genomics analysis

Cells from the three donors (RH1, RH2, and RH3) were used for these studies. Each cell line was processed as described above for flow cytometric analysis but cells were detached from plates using TrypLE select (Gibco 12563-029), an animal origin-free cell dissociation reagent, which was then inactivated by diluting with Hanks buffer without FCS. Cells were pelleted by centrifugation at 400 g for 7 min at 4 °C, and the supernatant removed. Cells were re-suspended and washed again with Hanks buffer then lysed using 700 μl QIAzol (Qiagen #79306). The RNA was isolated using a MiRNeasy Mini Kit (Qiagen #217004) and the on-column DNAse treatment was performed according to the manufacturer’s instructions (RNAse free DNase set; Qiagen #79254). RNA concentrations were measured using a Nanodrop reader. The RNA samples were processed by automated RNASeq-FastQ sequencing using the NEXTflex™ Rapid Illumina Directional RNA-Sequencer (BIOO Scientific, Austin, Texas, USA); for each sample, 300 ng of total RNA was processed using the NEXTflex™ Rapid Illumina Directional RNA-Seq Library Prep Kit (BIOO Scientific, Austin, Texas, USA). Briefly, the method selects poly-adenylated mRNA with coated beads and then converts them to strand-preserved cDNA (via dUTP) before the ligation of sequencing adapters and barcodes. After PCR amplification for 15 cycles the samples were quantified by a fluorescence assay before pooling in equimolar ratios for sequencing. The sample pool was sequenced by the Illumina Nextseq 500 sequencer using a High Output v2 (2 × 75 bp) paired-end sequencing kit ((Illumina, San Diego, USA)) as per the manufacturer’s instructions except that the loading concentrations were reduced by 30% to 0.9 pM. The data were analysed with de-multiplexed reads that were aligned (human hg38) using the TopHat aligner and the differential expression of transcripts was assessed using Cufflinks in Illumina’s Base-space analysis cloud.

### Statistical methods

All data analysis and graphical representations were performed using Microsoft Excel for Mac (Microsoft version 15.33) and Prism for Mac (version 7.0b, GraphPad Software Inc.). Parametric data were analysed using one-way analysis of variance (ANOVA), with Tukey’s multiple comparison test undertaken when significant differences in means were observed. Non-parametric data were analysed using the Kruskal-Wallis test of median values followed by Dunn’s multiple comparison test. Treated/non-treated groups were compared using the two-tailed Student’s *t* test followed by Mann-Whitney *U* tests. *P* values < 0.05 were considered statistically significant. For the genomic cDNA sequencing, analysis of statistical differences in gene levels in cells from the 24- and 48-h primed and non-primed MPC cultures were determined using the manufacturers’ software with *q* values < 0.045 being accepted as significant. However, for the majority of gene changes identified, statistical significance was observed at the *q* = 0.017 level.

## Results

### Kinetics of binding and uptake of PPS by MPCs in culture

**Fig. 1:**
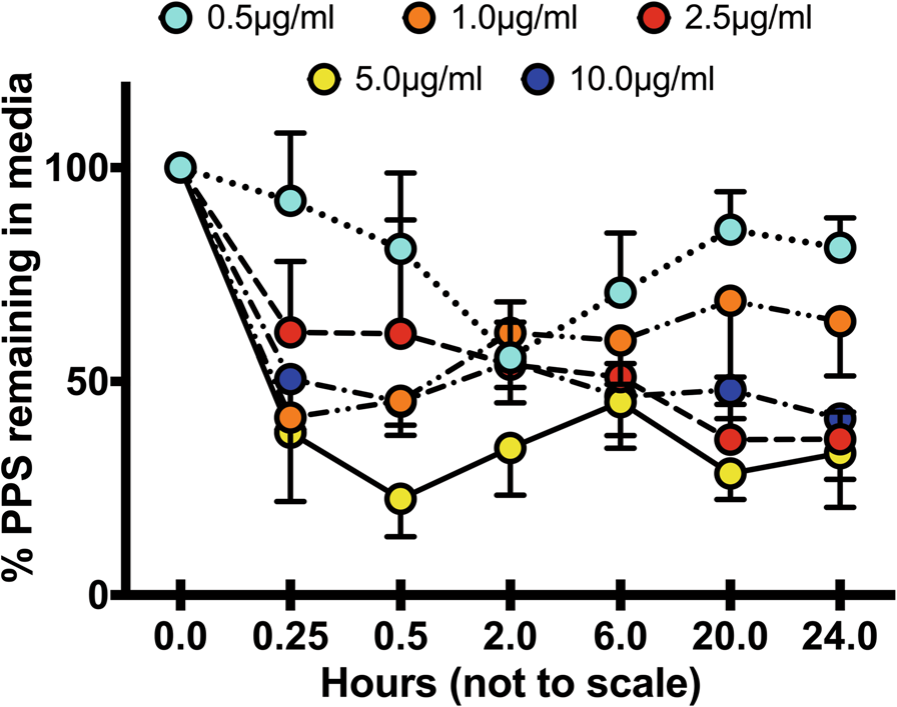
Kinetics of uptake of various concentrations of pentosan polysulfate (*PPS*) (0.5, 1.0, 2.5, 5, and 10 μg/ml/well) by 3.0 × 10^5^ MPCs/well maintained in monolayer cultures for 24 h. The concentration of PPS remaining in the culture media after 0.25, 0.50, 2, 6, 20, and 24 h was determined using ELISA. Note the rapid decline in PPS media concentrations within 2 h of culture followed by shedding of PPS into the media with the 0.5 and 1.0 μg/ml concentrations. The media concentration of 5.0 μg/ml exhibited the highest uptake by the MPCs over the 24-h culture period

The kinetics of binding and uptake of PPS by cultured MPCs when added to the media at concentrations of 0.5–10 μg/ml was monitored by the percentage decrease in their media levels over 24 h using the PPS ELISA. As shown in Fig. 1, all concentrations of PPS added to the culture media decreased over the first 0.5–2.0 h of incubation with MPCs. For media concentrations of 0.5 and 1.0 μg/ml PPS, this initial decline was followed by a partial release of PPS into the media over the subsequent 6–24 h (shedding period). However, for cultures spiked with 2.5, 5.0, or 10.0 μg/ml PPS, the reduced media levels were sustained over this period. Interestingly, cultures to which 5.0 μg/ml PPS had been added demonstrated the highest decline in media levels after 0.5 h and only released relatively small amounts over the subsequent 24-h period (Fig. 1). These observations suggest a rapid binding of PPS to cell surface heparin receptors, followed by a time- and concentration-dependent shedding and uptake by the MPCs over the 24 h of culture [28, 29]. Moreover, under the conditions used for these cultures, optimum uptake of PPS by MPCs was found to occur with a medium concentration of 5.0 μg/ml.

**Fig. 2:**
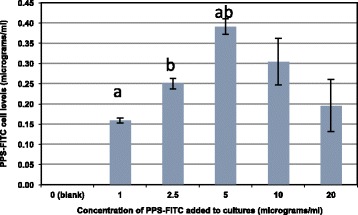
Concentration-dependent uptake of PPS-FITC by MPCs (2.5 × 10^5^ cells/well) determined using the fluorometric assay. Highest uptake was observed with PPS-FITC concentrations of 5.0 μg/ml which were significantly different to 1.0 and 2.5 μg/ml. ^a^ *p* < 0.004; ^b^ *p* < 0.012. *FITC* fluorescein isothiocyanate, *PPS* pentosan polysulfate

**Fig. 3:**
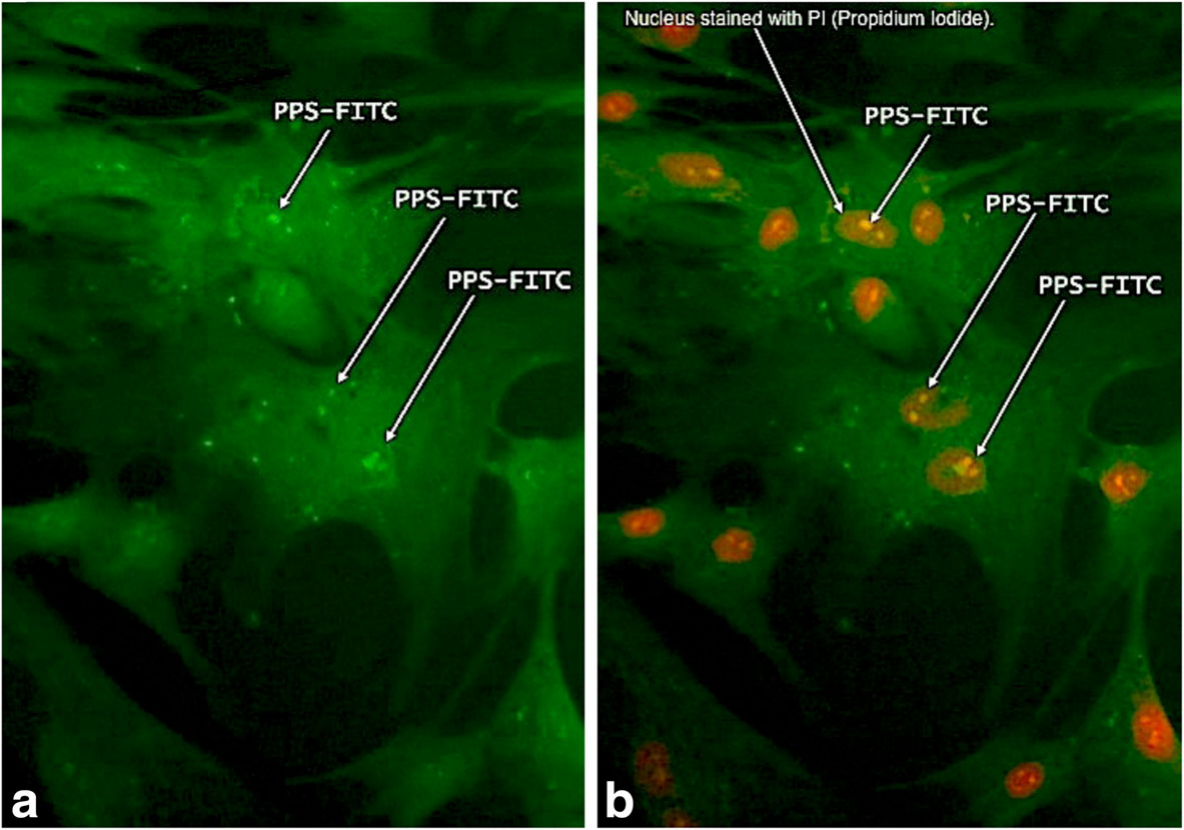
Fluorescence microscopy images of MPCs (6000/well) cultured with 2.5 μg/ml PPS-FITC for 24 h, fixed in Histochoice MB/ethanol and stained with propidium iodide. **a** A 2-s exposure image of the MPCs outlined by the low-level background autofluorescence and the higher emission arising from the PPS-FITC located within the cells (*arrows*). **b** The same field as **a**, but with 60-s exposure with excitation at 535 nm confirming the presence of PPS-FITC within the nucleus of the MPCs (*arrows*). *FITC* fluorescein isothiocyanate, *PPS* pentosan polysulfate

As the PPS ELISA was not sufficiently sensitive to evaluate the amounts of PPS associated with the MPCs following their removal from culture, we used the PPS-FITC preparation and a fluorometric assay to assess the amounts of PPS associated with the MPCs. This was coupled with fluorescence microscopy to identify the intra-cellular distribution of PPS over the indicated time points. The results of these studies are shown in Figs. 2 and 3. As is evident from Fig. 2, significantly higher levels of PPS-FITC were associated with the MPCs after 24 h of culture with 5.0 μg/ml than with 1.0 μg/ml (*p* < 0.004), 2.5 μg/ml (*p* < 0.012), or 20 μg/ml as a trend (*p* < 0.054). However, significant difference could not be demonstrated between media concentration of 5.0 and 10.0 μg/ml using the PPS-FITC fluorometric assay.

Qualitative studies of the interaction of PPS-FITC with MPCs using fluorescence microscopy together with co-staining of the preparations with the selective nucleus stain PI showed that, after 16 h of culture, the PPS-FITC was largely located within the nucleus of the cell (Fig. 3).

**Fig. 4:**
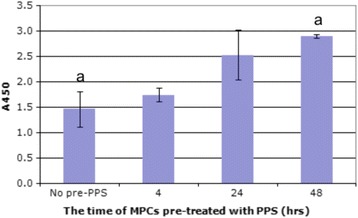
Proliferation of non-primed and pentosan polysulfate (*PPS*) (5.0 μg/ml)-primed mesenchymal progenitor cells (*MPCs*) over 4, 24, and 48 h determined using the Wst-8 assay kit. As no significant differences in proliferation were observed for the non-primed MPCs, the values for 4, 24, and 48 h were combined. Primed MPCs cultured for 48 h were significantly different from the pooled non-primed cultures (^a^ *p* < 0.028). *A450* absorbance at 450 nm

Although the kinetic and fluorometric studies on the uptake of PPS by MPCs suggested that with media concentrations of 5.0 μg/ml more than 50% of the agent was bound and internalised by the cells, the culture periods used never exceeded 24 h. A study was therefore undertaken to monitor MPC proliferation when the cells were cultured alone or after pre-incubation (priming) with 5.0 μg/ml PPS for 4, 24, and 48 h. The results of this study are shown in Fig. 4 where it is evident that MPCs primed with PPS increased proliferation after 48 h to a significantly higher extent than non-primed MPCs (*p* < 0.028). As an earlier study [21] had reported that co-cultures of MPCs with PPS promoted chondrogenic differentiation, we next investigated the biosynthesis of PGs of MPCs alone and after pre-culturing with PPS as described for the proliferation study.

**Fig. 5:**
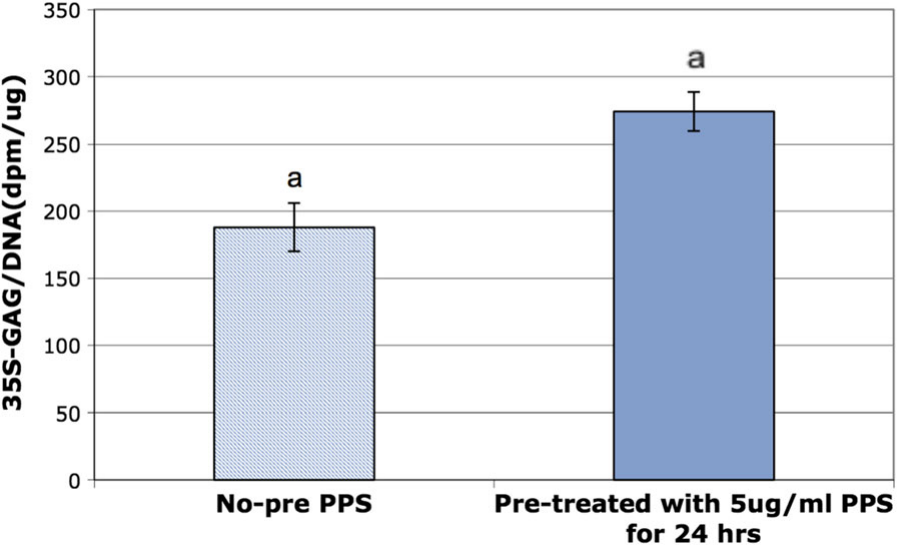
Biosynthesis of PGs determined by the incorporation of ^35^SO_4_ into their sulphated glycosaminoglycans (*35S-GAG*) in 24-h cultures of non-primed MPCs and MPCs primed with pentosan polysulfate (*PPS*). Primed MPCs synthesised 40% more PGs than non-primed MPCs after the 24-h culture. ^a^ *p* < 0.005

The results of this experiment are shown in Fig. 5 and demonstrate that the MPCs primed with PPS increased de novo PG biosynthesis to a greater extent than when MPCs were cultured alone (*p* < 0.005). Since the PPS priming process was known to promote MPC proliferation (Fig. 4), we normalized the incorporation of ^35^SO_4_ into the S-GAGs of the newly synthetized PG relative to cell numbers (DNA content).

**Fig. 6:**
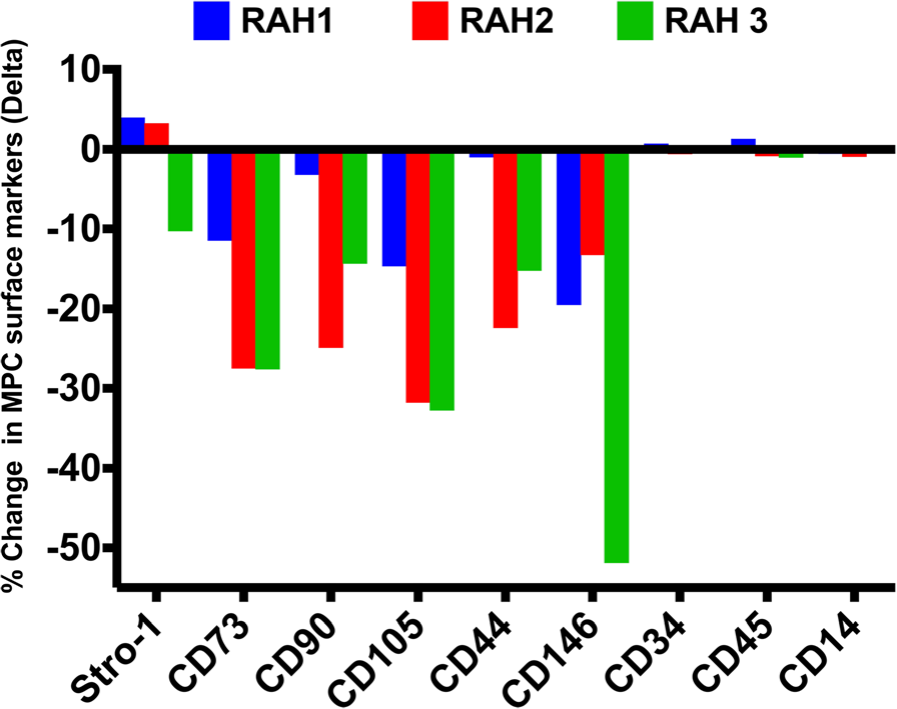
Graphical representation of the combined changes induced in mesenchymal progenitor cell (*MPC*) characteristic surface antigens expressed on cells from three donors (RAH1, RAH2, and RAH3) that had been cultured for 24 and 48 h with and without priming with PPS. The raw data obtained by flow cytometric analysis is shown in Additional file 1. Delta represents the total difference (as a percentage) for each donor between primed and non-primed MPC antigen values. *CD* cluster differentiation

In view of these findings, we next sought to determine, using flow cytometry, if the PPS priming process also induced changes in the MPC cell surface phenotypic antigens after culturing the primed and non-primed cells for 24 and 48 h. The results of these studies are shown in Fig. 6 and Additional file 1, where the net differences between primed and non-primed MPC antigen levels were calculated for each donor and expressed as their delta change. Figure 6 depicts graphically the total delta changes that occurred in surface antigen levels for each donor over the 24- and 48-h culture periods. As is shown, donors RAH2 and RAH3 exhibited patterns of changes with marked decreases in the CD73, CD90, CD105, and CD44 surface antigens of between 15–30%. However, expression of CD146 on MPCs from donor RAH3 declined by more than 50%. MPCs from donor RAH1 were found to be less responsive to the priming procedure but still exhibited the same pattern of decline in the characteristic MPC surface phenotype receptors. Interestingly, the STRO-1 marker used to isolate the MPCs from bone marrow aspirates was not markedly affected by the priming step; only donor RAH3 exhibited a 10% decrease, with the cells from the other two donors showing minimal change in expression of this antigen following the PPS priming procedure. The low levels of the hematopoietic and monocyte cell markers CD34, CD45, and CD14 were not affected by PPS priming, suggesting preservation of the mesenchymal cell lineage (Fig. 6).

Additional evidence to support the finding that priming of MPCs with PPS mediated altered gene expression by these cells was provided by isolating the RNA extracted from MPCs of the three donors after culturing for 24 and 48 h and undertaking RNASeq-FastQ sequencing. The results of this study are shown in Tables 2 and 3, which record the mean statistically significant gene changes for the three donors that were detected between their primed and non-primed MPCs after 24 and 48 h of culture. Using internet-based gene search engines, the proteins encoded by these genes are also identified in Tables 2 and 3. These datasets show that after the initial 24 h of culture only four genes were upregulated and 16 downregulated (Table 2) by the priming process. However, after 48 h 16/42 genes were upregulated and 26 downregulated.

**Table 2:** Gene expression changes induced by 24-h cultures of MPCs with 5.0 μg/ml PPS relative to identical cultures of non-primed MPCs

Gene	Fold change	Regulation	Primary functions^a^
ACTA2*	1.21	Down	Encoding actin-2 protein a member of the actin family which collectively are responsible for cell motility, structure and integrity.
ADAMTSL4	0.77	Down	Encodes the protein ADAMTS4 which lacks a C-terminal TS motif but when proteolytically processed generates the mature proteinase that degrades aggrecan, a major component of hyaline cartilage.
ANK1*	2.79	Up	Encoding the protein Ankyrin-1 a member of the Ankyrin family that play key roles in cell motility, activation, and proliferation.
COL11A1*	1.21	Down	Encoding one of the alpha chains of type XI collagen.
COL5A3*	2.22	Up	Encodes one of the alpha chains of type V collagen
COMP*	1.08	Down	COMP gene provides the instructions for making the COMP protein, an important regulatory component of the extracellular matrix.
DACT1	1.28	Down	Encodes a protein member of the Dapper family. It interacts with and positively regulates dishevelled-mediated signalling pathways during development and is an antagonist of beta-caterin.
ENPP1	0.58	Down	Encoded protein is type II transmembrane glycoprotein that cleaves a variety of substrates including phosphodiester bonds of nucleotides.
FLG*	1.82	Down	The FLG gene provides instructions for making the large protein profilaggrin.
GREM2	0.64	Down	Encodes a member of the BMP antagonist family likely by binding to BMPs
HSPB7*	1.56	Down	Encodes a member of the heat shock beta-7 protein family
LARGE	0.94	Down	Encodes members of the N-acetylglucosamine-l-transferase protein family responsible for glycosylation of glycoproteins and glycosphingolipids.
LMOD1	0.52	Down	Encodes the leicmodin 1 protein that has a putative membrane-spanning region and two types of tandemly repeat blocks.
LOXL4	0.64	Down	Encodes a member of the lysyl oxidase family essential for the biogenesis of crosslinks of matrix collagens and elastins.
LRRC15	0.76	Down	Encodes the leucine rich repeat containing 15 protein that constitute regions of the small proteoglycans.
MRVI1	0.83	Down	Encoding protein MRVI1, a substrate of cGMP-dependent kinase-1(PKG1).
SCUBE3	0.86	Down	Encodes Signal peptide-CUB and EGF-like Domain-containing Protein3.
SVIL	0.64	Up	Encodes the protein Supervillin which is tightly associated with actin filaments and plasma membranes.
SYNPO2	1.06	Down	Encodes Synaptodin 2-like protein, GO annotations include actin binding.
TM4SF1	0.72	Up	Encodes a member of the transmembrane 4 superfamily that mediate signal transduction in the regulation of development, activation, and growth.

^a^ From Gene Cards Human Gene Database Index, Weizmann Institute of Science, 234 Herzi Street, Rehovat 7610001, Israel

* Confirmed via alternative analysis (Star/DESeq)

*ADAMTS* a disintegrin-like metalloproteinase with thrombospondin motifs, *COMP* cartilage oligomeric matrix protein, *GO* gene ontology, *MPC* mesenchymal progenitor cell, *PPS* pentosane polysulfate

**Table 3:** Gene expression changes induced by 48-h cultures of MPCs with 5.0 μg/ml PPS relative to identical cultures of non-primed MPCs

Gene	Fold change	Regulation	Primary functions^a^
ABCA8	2.6	Up	The ABCC8 gene provides instructions for making the sulfonylurea receptor 1 (SUR1) protein. The SUR1is a subunit of the ATP-sensitive potassium (K-ATP) channel.
ABI3BP	0.9	Up	Encodes the ABI family member 3 (NESH) binding protein. GO annotations of this gene include heparin and collagen binding.
ACAN	0.8	Up	Encoding for the Aggrecan core protein, also known as cartilage-specific proteoglycan core protein (CSPCP) or chondroitin sulfate proteoglycan 1.
ASNS*	1.1	Down	The ASNS gene encodes the enzyme asparagine synthetase (EC 6.3.5.4 )
CACNA2D1	1.1	Up	Encoding calcium voltage-gated channel auxiliary subunit alpha2delta 1 that mediates calcium channel regulatory activity.
CBS	1.0	Down	Cystathionine β-synthase (CBS; l-serine hydro-lyase) adding homocysteine homocystinuria.
CD74	2.0	Up	HLA class II histocompatibility antigen gamma chain also known as HLA-DR antigens-associated invariant chain or CD74.
CHI3L1	1.5	Up	Chitinase-3-like protein 1, also known as YKL-40, is a secreted glycoprotein.
CNN1	0.7	Down	Encodes a matricellular protein also known as epididymis protein 1 that induces fibroblast senescence and has been reported to restrict fibrosis in cutaneous wound healing.
COMP	3.2	Down	COMP gene provides the instructions for making the COMP protein, an important regulatory component of the extracellular matrix.
CRISPLD2	0.8	Down	Cysteine rich secretary protein LCCL domain 2, exhibits significant LPS binding affinity.
DDIT4	0.7	Down	DNA damage inducible transcript 4 regulates cell growth, proliferation and survival via inhibition of the activity of the mammalian target of rapamycin complex 1 (mTORC1).
FLG	1.2	Down	The FLG gene provides instructions for making the large protein profilaggrin
FOSB	101.0^#^	up	FosB transgene is associated with the induction of the AP-1 complex. FosB interacts with Jun oncoproteins enhancing their DNA binding activity.
GGT5	1.8	Up	Encodes the gamma-glutamyl-transpeptidase protein family. After post-translational modification, the protein can convert Leukotriene C4 to Leukotriene D4.
FST	0.7	Up	Encodes Follistatin, also known as activin-binding protein. Its primary function is the binding and bioneutralization of members of the TGF-β superfamily.
GHRL	84.6^#^	Down	Encodes Growth Hormone protein releasing peptides protein.
HIST2H3A	99.4^#^	Up	Encodes Histone Cluster 2, H3a protein. Histones play a central role in transcription regulation, DNA repair, and regulation of gene expression.
HMGA1	0.6	Up	Encodes High Mobility Group AT-Hook 1 that regulates inducible gene transcription.
HMGA2	0.8	Up	Encodes High Mobility Group AT-Hook 2, a protein coding gene which contains structural DNA binding regions that may act as transcriptional regulating factors.
IGF2	1.6	Up	Encodes the Insulin-Like Growth Factor 2 protein family that play essential roles in growth and development.
LARGE	0.8	Down	Encodes members of the N-acetylglucosaminyltransferase protein family responsible for glycosylation of glycoproteins and glycosphingolipids.
LRRC15	1.8	Down	Gene encoding Leucine Rich Repeat Containing 15 Proteins. GO annotations related to this gene include collagen binding and laminin binding.
MASP1	1.9	Down	Gene encoding mannan binding lectin serine peptidase 1 that regulates the lectin pathway of complement activation.
METTL7A	1.4	Up	Encodes Methyltransferase Like 7A protein. GO annotations related to this gene include methyltransferase activity and S-adenosylmethionine-dependent methyltransferase activities.
MTHFD2*	0.9	Down	Encodes methylenetetrahydrofolate dehydrogenase (NADP + dependent) 2 enzyme, activities that allows binding of NAD.
NFATC2	1.8	Down	Encodes for Nuclear factor of activated T-Cells 2 protein that resides in the cytosol and only translocate to the nucleus upon T-cell receptor stimulation where it becomes a member of the nuclear factors of the activated T-cell transcriptional complex.
OLFML2A	1.7	Up	Encodes for Olfactomedin-Like 2A protein. GO annotations related to this gene include protein homodimerization activity and extracellular matrix binding.
PAMR1	1.2	Up	Encoding peptidase domain containing associated with the muscle regeneration 1
PHGDH*	1.1	Down	Encoding D-3-phosphoglycerate dehydrogenase (catalyses the transition of 3-phosphoglycerate into 3-phosphohydroxypyruvate, which is the committed step in the phosphorylated pathway of l-serine biosynthesis. It is also essential in cysteine and glycine biosynthesis.
PIM1	0.8	Down	Encoding Proto-oncogene serine/threonine-protein kinase Pim-1. It plays a role in signal transduction in blood cells, contributing to cell proliferation and survival.
POM121L9P	2.9	Up	This gene encodes a transmembrane protein that localizes to the inner nuclear membrane and forms a core component of the nuclear pore complex, which mediates transport to and from the nucleus.
PSAT1	1.8	Down	Encoding spermidine/spermidine Ni-acetyltransferase 1 which is a rate limiting enzyme in the catabolic pathway of polyamine metabolism.
PTX3	1.2	Up	Encoding pentraxin-related protein PTX3 also known as TNF-alfa induced protein 5. The expression of this protein is induced by inflammatory cytokines in response to inflammatory stimuli in several mesenchymal and epithelial cell types. It also plays a role in angiogenesis and tissue remodelling.
SLC38A1*	0.9	Down	Encoding sodium-coupled neutral amino acid transporter 1, production of which plays an essential role in the uptake of nutrients, energy production, chemical metabolism, and detoxification.
SLC7A11	1.3	Down	Encoding solute carrier family 7 member 11 protein that is highly specific for cystein and glutamate amino acids.
SLC7A5	0.8	Up	Encoding solute carrier family 7 member 5 protein that transports large neutral amino acids.
SVIL	0.7	Up	Encodes Supervillin. The gene product is tightly associated with both actin filaments and plasma membranes, suggesting a role as a high-affinity link between the actin membranes, suggesting a role as a high-affinity link between the actin and the membrane.
THSD4	0.5	Up	Encoding thrombospondin type-1 domain containing protein 4. The thrombospondin family members are adhesive glycoproteins that mediate cell-to-cell and cell-to-matrix interactions.
TMEM200A	0.8	Down	Encoding transmembrane protein 200A
TPPP3	122.5^#^	Down	Tubulin polymerisation promoting protein family member 3 a protein encoding gene. GO annotations of this gene include tubulin binding.

^a^From Gene Cards Human Gene Database Index, Weizmann Institute of Science, 234 Herzi Street, Rehovat 7610001, Israel

*Confirmed via alternative analysis (Star/DESeq)

^#^Fold-change overestimated due to ‘zero’ measurement in one sample

*ADAMTS* a disintegrin-like metalloproteinase with thrombospondin motifs, *COMP* cartilage oligomeric matrix protein, *GO* gene ontology, *MPC* mesenchymal progenitor cell, *LPS* lipopolysaccharide, *PPS* pentosane polysulfate, *TGF* transforming growth factor

## Discussion

This study has shown that priming of MPCs with PPS results in the initial binding of the drug to the cell surface receptors accompanied by partial shedding, and then internalization and migration to the cell nucleus where it influenced gene and protein expression. The extent of changes induced in MPC cell surface markers by the PPS priming step for the three donors was found to be variable (Fig. 6). Indeed, differences in gene expression by bone marrow-derived MSCs from different donors have been previously reported as a potential problem for their routine application in clinical practice [30]. This inter-donor variability has also been attributed to a variety of other factors, including the inherent heterogeneity of the MSC populations isolated from different individuals, the duration of their culture expansion, and the period and nature of their storage [31–33]. The MPCs used in the present study were all within the age range of 20–35 years, were selected on the basis of their expression of STRO-1, and were subjected to similar culture and storage conditions to minimize inter-donor cell variability. Nevertheless, the magnitude of change in MPC surface marker expression induced by the PPS priming step for these three donors was found to be quite variable, suggesting that individual genetic variations may represent a dominant role. However, apart from STRO-1, the markers CD73, CD90, CD105, CD44, and CD146 were all observed to decline following PPS priming of the cells.

Human MSC monolayer cultures incubated with transforming growth factor (TGF)-beta for 7 days have been reported to undergo a similar downregulation of the surface antigens CD44, CD90, and CD105, a finding that was interpreted to signal an early phase of their de-differentiation to the chondrogenic phenotype [34]. We also observed a strong decline in CD146 antigen presentation on PPS priming, particularly for MPCs isolated from donor RAH3. The transmembrane protein CD146 is receptor highly expressed by endothelial cells [35] and on the surface of perivascular cells, which have recently been proposed as the source of MSCs within the perivascular niche of bone marrow [36]. Moreover, a recent study has provided compelling evidence that CD146 is a high-affinity netrin-1 receptor on endothelial cells [37]. Netrin-1 is a neuronal guidance molecule that promotes angiogenesis and vascular development of the endothelium following interaction with CD146 [36, 37]. In addition, expression of CD146 is associated with populations of human MPCs that promote the establishment of bone marrow elements, and enhance osteogenic differentiation and bone deposition when these cells are implanted subcutaneously into immune-deficient mice [38]. The present observation that CD146 expression by MPCs was markedly downregulated by PPS priming would therefore be consistent with our previous observations of reduced osteogenesis of MPCs when cultured or co-formulated with this agent in vitro [21] and in vivo [22–24].

Although many of the functions of the proteins encoded by the genes identified by RNA sequencing analysis could not be obviously assigned, the changes in the genes encoding the aggrecan core protein, IGF2, alpha chain type V collagen, FosB transgene, COMP, the proteinase ADAMTS4, and type II collagen alpha chains provided are consistent with increased chondrogenic differentiation of MPCs. For example, aggrecan core protein is necessary for the biosynthesis of PGs [39] and its upregulation is consistent with the known elevation of their biosynthesis by MPCs after PPS priming. The down regulation of the ADAMTSL4 gene could also be considered as beneficial for the deposition of a cartilaginous matrix as the protein it encodes is responsible for the degradation of PGs [39]. In addition, the upregulation of type V collagen could be significant as this protein is a contributor to the assembly of collagen fibres during cell growth and matrix assembly [40]. On the other hand, the downregulation of the COMP genes was unexpected since this protein is an abundant component of the cartilage extracellular matrix. However, studies with human MSCs have shown that enhancement of COMP gene expression did not increase the transcript levels of the chondrogenic markers Sox9 or aggrecan, suggesting that the role of COMP in matrix formation occurs at the post-transcriptional level [41]. Notably, the IGF2 gene was found to be strongly upregulated. As the proteins encoded by this gene play significant roles in the growth, differentiation, and survival of connective tissue cells, including articular cartilage [42], its elevation is consistent with the present study and our previous report on MPC chondrogenesis mediated by PPS [21]. The RNASeq-FastQ sequencing data also indicated that the FosB transgene was strongly upregulated by the priming process. Numerous studies have shown that the Fos genes are involved in the formation of heterodimeric complexes with members of the jun family of proto-oncogenes (c-jun, junB, jun D) to form the AP-I promotor complex required for gene transcription [43]. Following binding to consensus sequences in the regulatory regions of DNA, the Fos-Jun/AP1 complex mediates transcription pathways responsible for critical cell functions, including differentiation and turnover of the extracellular matrix [44].

A related sulphated glycosaminoglycan, heparin, is known to bind and interact with a variety of cells where it also localizes in the nucleus and modifies gene expression [31, 32, 45–48]. Moreover, heparin has been used at low concentrations (<200 ng/ml) as a supplement for the culture expansion of embryonic stem cells [49, 50] and MSCs [51]. However, in a recent study which used human bone marrow-derived MSCs [52], it was demonstrated that when serial cultures of these cells were supplemented with heparin at a concentration equivalent to that used in the present study (500 μg/ml), cell growth was strongly retarded and MSC morphology and genetic expression modified to a senescent phenotype. These conflicting findings may be explained by the structural differences between these two polymers.

**Fig. 7:**
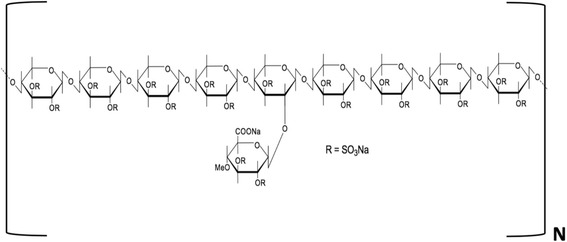
Structural formula of the repeating unit of the poly-dispersed PPS. On average, a single sulphated 4-O-methyl-glucopyranosyluronic acid ring is attached laterally via an oxygen linkage to the 2 position of every sulphate-esterified 9–10th xylanopyranose unit of the polymer. From the molecular weight distribution of 1800–17,000 Da determined by size exclusion chromatography [44], N can be estimated as 0.5–6.0

Like heparin, PPS is a poly-anion, but is not a glycosaminoglycan since it has a backbone structure consisting of repeating beta-d-xylanopyranose units to which a methyl glucopyranosyluronic acid ring is attached laterally every 9–10 xylanopyranoses units (Fig. 7). The xylanopyranose backbone required for the synthesis of PPS is extracted from Beech wood (*Fagus sylvatica*) hemi-cellulose, is first sulphate-esterified, and then fractionated to obtain the required molecular size. This semi-synthetic process affords a water-soluble poly-dispersed pharmaceutical preparation with a weight average molecular weight (MW) of 5700 Da and a high negative charge conferred by the large number of sulphate ester groups localised along its xylanopyranose backbone [53].

In contrast, native heparin is a structurally heterogeneous biopolymer that consists essentially of variably spaced repeating units of either 2-O-sulphated iduronic acid and 6-O-sulphated and N-sulphated glucosamine sugar rings linked glycosidically [54]. Commercially available heparin is more poly-dispersed than PPS with an averaged molecular weight ranging between 3000 to 30,000 Da [53] but is the most highly sulphated naturally occurring glycosaminoglycan with 2.7 sulphate groups/disaccharide unit [54]. However, its charge density is less than that of PPS which on average contains 3–4 sulphate groups/disaccharide unit (Fig. 7).

Notwithstanding these significant molecular, charge, and conformational differences, PPS, because of its poly-anionic structure, does exhibit some heparin-like pharmacological activities. Although it is a weaker anti-coagulant than heparin, PPS is a strong fibrinolytic and lipolytic agent [52, 54]. These pharmacological activities resulted in its original clinical applications in the 1950s for the treatment of thrombotic and arteriosclerotic vascular disease [55]. However, over the intervening years, PPS has been shown to be effective for the management of more diverse medical indications, including interstitial cystitis [56], soft tissue inflammation [57], osteoarthritis [58–60], and Ross River Virus-related arthropathies [61].

In our earlier in-vitro studies, MPCs were cultured with PPS at various concentrations including 5.0 μg/ml, but for up to 10 days [21]. With the longer incubation periods, gene expression of Sox-9 and Aggrecan by MPCs was not significantly elevated relative to MPCs alone until day 7. In addition, expression of type II collagen was not significantly increased until day 10, when type X collagen, RUNX2, and Noggin gene expression was also suppressed [21]. These earlier RNA studies suggest that the present protocol of 24-h priming of MPC with PPS followed by maintaining cultures for up to 48 h prior to determination of gene expression may have been too short to establish the lifetime of genetic modifications. We therefore acknowledge that the maintenance of our PPS-primed MPC cultures for only 48 h represents a limitation of the present study. However, using an ovine model of disc degeneration induced by lumbar microdiscectomy we have demonstrated that PPS-primed MPCs when embedded in biodegradable collagen sponges implanted into the degenerate discs promoted the deposition of higher levels of proteoglycans and tissue repair after 6 months, compared with the injured disc injected with non-primed MPCs [62]. We consider that this in-vivo study supports our proposition that PPS-primed MPCs retained their modifying effects on gene and protein expression beyond the 48-h experimental period used in the present study.

## Conclusions

These studies have shown that pre-incubation of MPCs with 5.0 μg/ml PPS for only 24 or 48 h was sufficient to invoke significant changes in their gene signature and protein expression consistent with enhanced proliferation and differentiation to the chondrogenic phenotype. The PPS priming step was undertaken at the penultimate phase of MPC culture expansion, a procedure that eliminated the necessity of combining the required quantities of the two agents at the time of clinical application and thereby eliminating the possibility that ‘free’ PPS was co-administered with the progenitor stem cells. Furthermore, from the results of the present study, together with the positive outcome of our animal model study [62], we conclude that pre-culturing of MSCs with agents such as PPS could provide an alternative method for reprogramming these cells to promote their differentiation towards a targeted phenotype that may be required for a specific medical indication, rather than their co-administration with agents that may independently be associated with undesirable side effects.

## Additional file

Additional file 1: (A–E) Surface antigen expression of MPCs derived from three donors (RAH1, RAH2, and RAH) when cultured for 24 and 48 h with and without priming with 5.0 μg/ml PPS. Delta change represents the percentage change in antigen levels mediated by the PPS priming step. (DOCX 18 kb)

## Abbreviations

ADAMTS: A disintegrin-like metalloproteinase with thrombospondin motifs
AP: Alkaline phosphatase
BSA: Bovine serum albumin
CD: Cluster differentiation
COMP: Cartilage oligomeric matrix protein
CPC: Cetyl pyridinium chloride
DMEM: Dulbecco’s modified Eagle’s media
ELISA: Enzyme-linked immunosorbent assay
FBS: Fetal bovine serum
FCS: Fetal calf serum
FITC: Fluorescein isothiocyanate
GAG: Glycosaminoglycan
IGF: Insulin-like growth factor
MPC: Mesenchymal progenitor cell
MSC: Mesenchymal stem cell
PBS: Phosphate-buffered saline
PG: Proteoglycan
PI: Propidium iodide
PNP: Para-nitrophenyl phosphate
PPS: Pentosan polysulfate
TBA: Tetrabutyl ammonium
TGF: Transforming growth factor
