# Supplementary material for: Pentosan polysulfate binds to STRO-1+ mesenchymal progenitor cells, is internalized, and modifies gene expression: a novel approach of pre-programing stem cells for therapeutic application requiring their chondrogenesis
Source: Stem Cell Res Ther. 2017 Dec 13;8:278. doi: 10.1186/s13287-017-0723-y (PMC5729458; doi:10.1186/s13287-017-0723-y)
Supplement: Additional file 1: — (A–E) Surface antigen expression of MPCs derived from three donors (RAH1, RAH2, and RAH) when cultured for 24 and 48 h with and without priming with 5.0 μg/ml PPS. Delta change represents the percentage change in antigen levels mediated by the PPS priming step. (DOCX 18 kb) [file 13287_2017_723_MOESM1_ESM.docx]

**Additional file A - E: Surface antigen expression of MPC derived from 3 donors RAH1, RAH2 and RAH3 when cultured for 24 and 48 hours with and without priming with 5.0 µg/ml** **PPS. Delta Change represents the % change in antigen levels mediated by the PPS priming step.**

**A: Donor RAH1**

| **24 hour Cultures** | **Donor RAH1** | **Donor RAH1** |  |
| --- | --- | --- | --- |
|  | **MPC alone %** | **MPC + PPS %** | **Delta Change** |
| **Stro-1** | 22.0 | 23.9 | 1.9 |
| **CD73** | 93.3 | 80.8 | -12.5 |
| **CD90** | 96.0 | 85.2 | -10.8 |
| **CD105** | 79.3 | 65.8 | -13.5 |
| **CD44** | 98.9 | 97.5 | -1.4 |
| **CD146** | 99.3 | 83.9 | -15.4 |
| **CD34** | 0.038 | 0.10 | 0.062 |
| **CD45** | 1.65 | 1.68 | 0.03 |
| **CD14** | 0.066 | 0.029 | -0.037 |

**B: Donor RAH1**

| **48 hour Cultures** | **Donor RAH1** | **Donor RAH1** |  |
| --- | --- | --- | --- |
|  | **MPC alone %** | **MPC + PPS %** | **Delta Change** |
| **Stro-1** | 17.1 | 19.2 | 2.1 |
| **CD73** | 98.0 | 99.0 | 1 |
| **CD90** | 91.5 | 99.1 | 7.6 |
| **CD105** | 98.8 | 97.6 | -1.2 |
| **CD44** | 98.8 | 99.3 | 0.5 |
| **CD146** | 92.4 | 88.3 | -4.1 |
| **CD34** | 0.0 | 0.29 | 0.29 |
| **CD45** | 0.33 | 1.63 | 1.3 |
| **CD14** | 0.047 | 0.059 | 0.012 |

**C: Donor RAH2**

| **24 hour Cultures** | **Donor RAH2** | **Donor RAH2** |  |
| --- | --- | --- | --- |
|  | **MPC alone %** | **MPC + PPS %** | **Delta Change** |
| **Stro-1** | 14.9 | 12.7 | -2.2 |
| **CD73** | 97.8 | 96.9 | -0.9 |
| **CD90** | 98.1 | 98.8 | 0.7 |
| **CD105** | 96.1 | 84.3 | -11.8 |
| **CD44** | 97.2 | 99.4 | 2.2 |
| **CD146** | 96.2 | 99.3 | 3.1 |
| **CD34** | 0.0 | 0.18 | 0.18 |
| **CD45** | 1.72 | 1.34 | -0.38 |
| **CD14** | 0.61 | 0.046 | -0.564 |

**D: Donor RAH2**

| **48 hour Cultures** | **Donor RAH2** | **Donor RAH2** |  |
| --- | --- | --- | --- |
|  | **MPC alone %** | **MPC + PPS %** | **Delta Change** |
| **Stro-1** | 15.9 | 21.4 | 5.5 |
| **CD73** | 83.0 | 56.4 | -26.6 |
| **CD90** | 48.5 | 22.9 | -25.6 |
| **CD105** | 92.1 | 72.1 | -20 |
| **CD44** | 92.9 | 68.3 | -24.6 |
| **CD146** | 87.0 | 70.6 | -16.4 |
| **CD34** | 0.27 | 0.0 | -0.27 |
| **CD45** | 0.22 | 0.0 | -0.22 |
| **CD14** | 0.21 | 0.0 | -0.21 |

**E: Donor RAH3**

| **24 hour Cultures** | **Donor RAH3** | **Donor RAH3** |  |
| --- | --- | --- | --- |
|  | **MPC alone %** | **MPC + PPS %** | **Delta Change** |
| **Stro-1** | 26.9 | 19.7 | -7.2 |
| **CD73** | 97.3 | 80.8 | -16.5 |
| **CD90** | 99.6 | 92 | -7.6 |
| **CD105** | 98.7 | 76.1 | -22.6 |
| **CD44** | 99.8 | 99.6 | -0.2 |
| **CD146** | 95.2 | 78.3 | -16.9 |
| **CD34** | 0.12 | 0.11 | -0.01 |
| **CD45** | 1.44 | 0.39 | -1.05 |
| **CD14** | 0.2 | 0.13 | -0.07 |

**E: Donor RAH3**

| **48 hour Cultures** | **Donor RAH3** | **Donor RAH3** |  |
| --- | --- | --- | --- |
|  | **MPC alone %** | **MPC + PPS %** | **Delta Change** |
| **Stro-1** | 19.3 | 16.2 | -3.1 |
| **CD73** | 89 | 77.9 | -11.1 |
| **CD90** | 68.2 | 61.4 | -6.8 |
| **CD105** | 96.5 | 86.3 | -10.2 |
| **CD44** | 98.5 | 83.5 | -15.0 |
| **CD146** | 84.1 | 49.1 | -35.0 |
| **CD34** | 0.0 | 0.0 | 0.00 |
| **CD45** | 0.0 | 0.059 | 0.059 |
| **CD14** | 0.037 | 0.11 | 0.073 |
